# Supplementary figures and images for: Induction of neutralizing antibodies against SARS-CoV-2 variants by a multivalent mRNA-lipid nanoparticle vaccine encoding SARS-CoV-2/SARS-CoV Spike protein receptor-binding domains in mice
Source: PLoS One. 2024 Apr 18;19(4):e0300524. doi: 10.1371/journal.pone.0300524 (PMC11025929; doi:10.1371/journal.pone.0300524)

Supplementary Figure 1

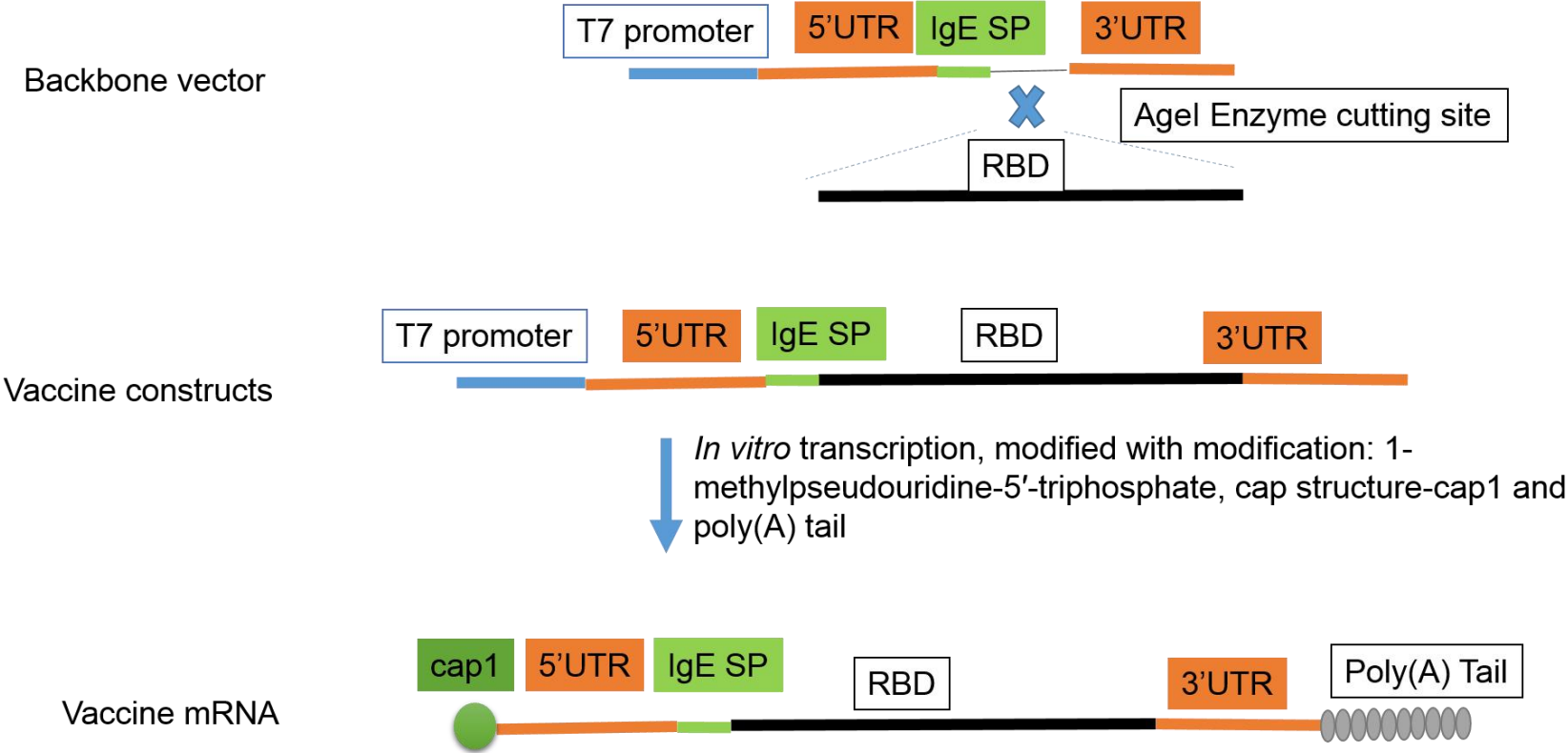

Supplement: S1 Fig — Design of mRNA constructs. (PDF) [file pone.0300524.s001.pdf]

Supplementary Figure 2

Anti-RBD

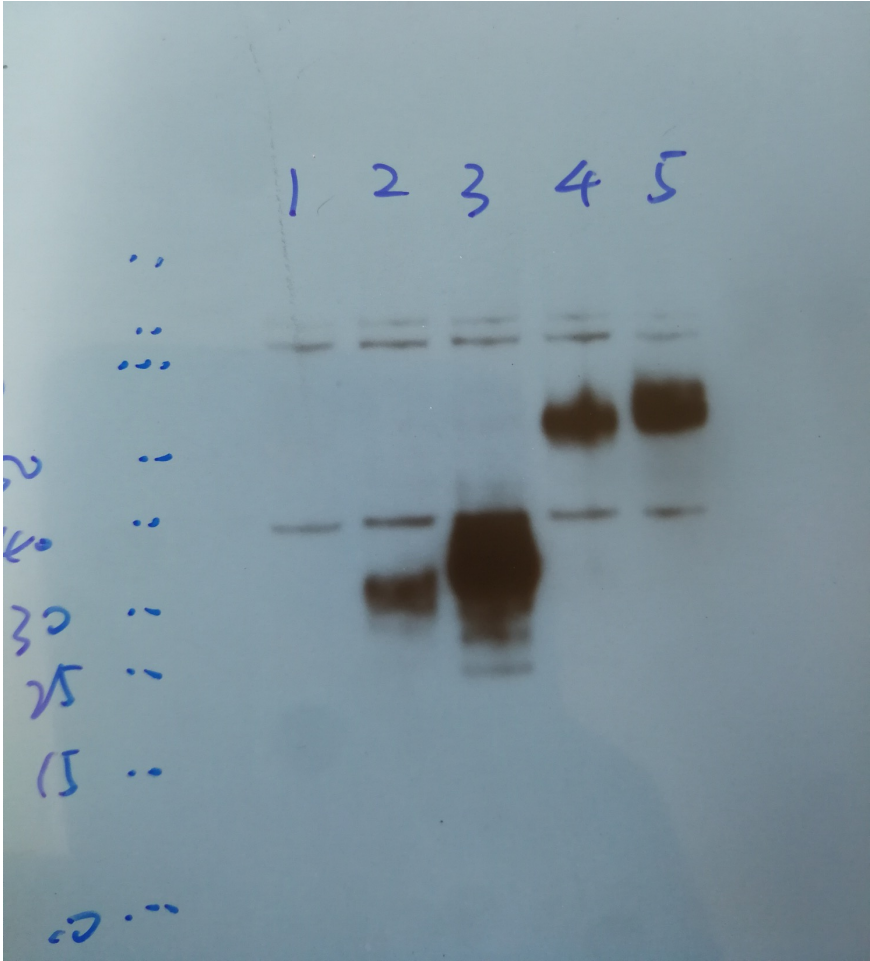

Anti-GAPDH

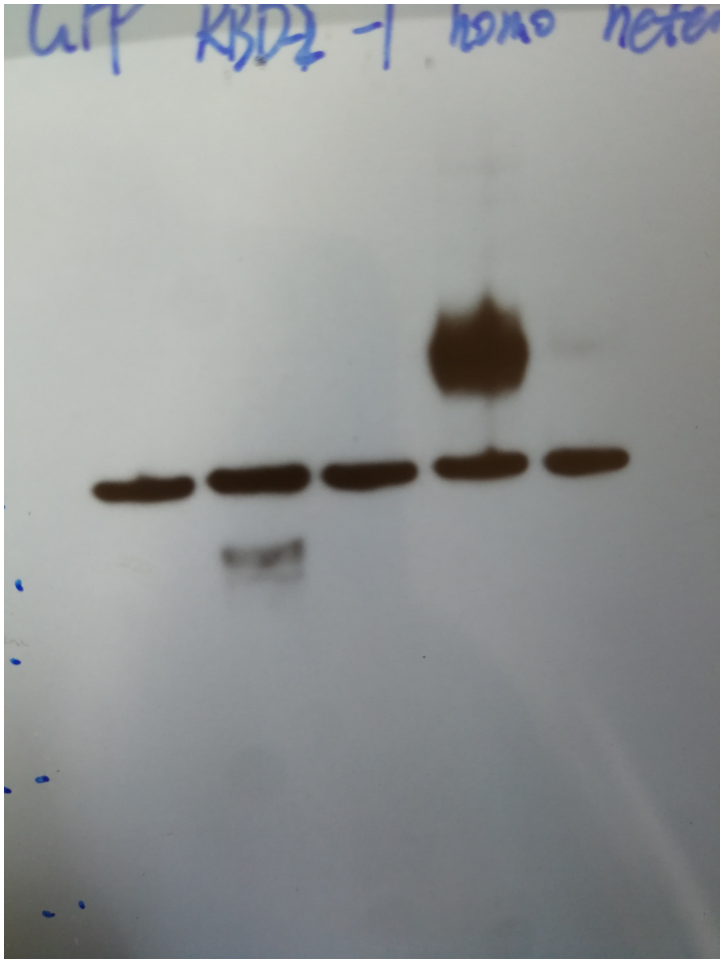

Supplement: S2 Fig — (PDF) [file pone.0300524.s002.pdf]
